# Model of the external force field for the protein folding process—the role of prefoldin

**DOI:** 10.3389/fchem.2024.1342434

**Published:** 2024-03-26

**Authors:** Irena Roterman, Katarzyna Stapor, Leszek Konieczny

**Affiliations:** ^1^ Department of Bioinformatics and Telemedicine, Jagiellonian University–Medical College, Krakow, Poland; ^2^ Department of Applied Informatics, Faculty of Automatic, Electronics and Computer Science, Silesian University of Technology, Gliwice, Poland; ^3^ Chair of Medical Biochemistry, Jagiellonian University–Medical College, Krakow, Poland

**Keywords:** protein folding, prefoldin, co-chaperone, tubulin, actin, chaperone, *in silico* analysis

## Abstract

**Introduction:** The protein folding process is very sensitive to environmental conditions. Many possibilities in the form of numerous pathways for this process can—if an incorrect one is chosen—lead to the creation of forms described as misfolded. The aqueous environment is the natural one for the protein folding process. Nonetheless, other factors such as the cell membrane and the presence of specific molecules (chaperones) affect this process, ensuring the correct expected structural form to guarantee biological activity. All these factors can be considered components of the external force field for this process.

**Methods:** The fuzzy oil drop-modified (FOD-M) model makes possible the quantitative evaluation of the modification of the external field, treating the aqueous environment as a reference. The FOD-M model (tested on membrane proteins) includes the component modifying the water environment, allowing the assessment of the external force field generated by prefoldin.

**Results:** In this work, prefoldin was treated as the provider of a specific external force field for actin and tubulin. The discussed model can be applied to any folding process simulation, taking into account the changed external conditions. Hence, it can help simulate the *in silico* protein folding process under defined external conditions determined by the respective external force field. In this work, the structures of prefoldin and protein folded with the participation of prefoldin were analyzed.

**Discussion:** Thus, the role of prefoldin can be treated as a provider of an external field comparable to other environmental factors affecting the protein folding process.

## 1 Introduction

Prefoldins are categorized as chaperone proteins, whose presence prevents the folding chain from adopting a structure unsuitable for performing a specific biological activity. Prefoldin interacts with the folding chain by imposing a specific transient structuring, thereby eliminating numerous other pathways that would lead to misfolding (Vainberg et al., 1998; DAmaro et al., 2008; Sahlan et al., 2018). Prefoldin is not directly involved in the folding process. Its role is limited to chain transport to the chaperonin. In the environment imposed by chaperonin, the folding protein obtains the expected final form that guarantees biological function (Hansen et al., 1999). The role of prefoldin, therefore, is to prevent the early acquisition of an inappropriate structure before the folding protein reaches the conditions for folding, leading to the expected functional structural form within the chaperonin. Thus, the presence of prefoldin eliminates the possible impact of the aqueous environment on the formation of the protein structure. Prefoldins are also referred to as co-chaperonins due to their formation of a short-lived complex with a chaperonin, mainly of the Hsp60 group (Siegert et al., 2000). The role of prefoldin is estimated to be of broad significance in stabilizing proteostasis by providing a balance between protein synthesis and the acquisition by proteins of the appropriate structure, including that prepared for the construction of an appropriate complex with other proteins (Arranz et al., 2018). Operating in the cytoplasmic environment, prefoldin has also been identified in the nucleus environment mainly in plants, yeast, and metazoa. In light of these studies, a role for prefoldin is also assumed as a coordinator in gene regulation and cytoplasmic environment process relationships (Millán-Zambrano and Chávez, 2014). A multi-subunit structure called AQosome (particle for the arrangement of quaternary structure), which interacts with chaperonins including Hsp90, Hsp70, and CCT, has also been linked to the activity of prefoldins (Lynham and Houry, 2018). The dysfunction of prefoldins links with the misfolding phenomena also related to neurodegenerative processes and tumor development (Yadav et al., 2019; Liang et al., 2020; Tahmaz et al., 2022). Prefoldin activity, or rather the lack of it, links with processes leading to amyloid formation in the case of Aβ proteins and α-synuclein in particular (Sörgjerd et al., 2014). In conclusion, the role of prefoldins, along with the entire group of chaperones, is described as critical to the functioning of the entire cellular machinery (Ohtaki et al., 2010; Gstaiger et al., 2003; D’Amaro et al., 2008; Gestaut et al., 2019). The object of the present analysis is prefoldin (PFD), the structure of which is available in the form of complexes with the chaperonin TRiC/CCT. Available structures include five different classes of this complex (Vainberg et al., 1998).

The experiments performed on prefoldin action deliver highly valuable information concerning its dependence on external conditions (temperature, ionic strength, and osmotic stress) (Blanco-Touriñán et al., 2021). Experimental approaches to study the biomolecular interactions driven under force, such as single-molecule force spectroscopy (Lostao et al., 2023) or optical tweezers (Bustamanta et al., 2020), highlight the potential limitations to these techniques as the complexity of calibration steps prior the data acquisition.

The analysis presented here is limited to assessing the structural diversity of prefoldin as a representative of co-chaperones—proteins responsible for preventing the mis-complexation of protein chains before an opportunity develops for the structuring expected for structures with biological activity (Vainberg et al., 1998). The prefoldin under consideration is built by six chains—a hetero-hexamer—where a single chain shows structuring classified according to CATH (Cathdb, 2021; https://www.cathdb.info/ accessed 1 Dec 2022; Sillitoe et al., 2021) as 1.10.287.370 is mainly the alpha orthogonal bundle. The hetero-hexamer arrangement is referred to as the jellyfish form (Lim et al., 2018). The five classes of the TRiC/CCT complex available in PDB (Laskowski and Thornton, 2021) allow the assessment of the structural diversity of PFD involved in the process of transporting the folding protein into the TRiC/CCT interior. The relationship of the PFD to the TRiC is referred to as a pivot. Class 2, which represents the state referred to as “latched,” is considered the initial state of the folding protein transport process into TRiC. Class 6 is referred to as “engaged” and is viewed last. The remaining available classes 3–5 structures are referred to as intermediates. The classification quoted here follows the interpretation of experimentally observed forms of complexes presented by Vainberg et al. (1998). This work is an attempt to assess the structure of prefoldin as a provider of a specific external force field for the transported protein. The aim is to prevent the structuring that could take place in an aqueous environment.

In the modified version of the fuzzy oil drop (FOD) model, the FOD-modified model quantitatively assesses the degree to which the structure of a relevant protein differs against the structuring possibly generated by the aqueous environment. It is assumed that the water environment induces the structuring of the folding protein, according to the micellization model (Konieczny et al., 2006; Banach et al., 2020). In other words, in the folding protein, a polar water environment induces the exposure of polar residues on the surface with a parallel centralization of hydrophobic residues. Thus, the hydrophobic core is isolated from the polar surroundings. The hydrophobicity distribution generated in this way within the protein is mapped by a 3D Gaussian function. A comparison of the idealized hydrophobicity distribution (full micellization) with that observed in the protein (inter-amino acid residues interactions) allows the assessment of differences assumed to be the result of the presence of a specific environment distinct from water. This degree of modification is taken as an indicator of the specificity of the external force field actively involved in the folding process. Hydrophobicity distribution in proteins representing the structure not requiring any modification of the water environment may be expressed by the 3D Gaussian function (full micellization). Such proteins were identified in the following groups of proteins: downhill, fast-folding, ultra-fast-folding, and in the group of antifreeze type II proteins (Banach et al., 2020). Moreover, it has been shown that the vast majority of domains present in proteins—units defined as folding independently of the rest of the protein—show structuration based on the distribution of hydrophobicity expressed by the 3D Gaussian function (Sałapa et al., 2012). Membrane proteins are a particular example where the change in the environment is radical. By taking reference to the FOD-M model, the effect of the hydrophobic membrane environment on the structuration of membrane proteins was quantified (Roterman et al., 2021a; Roterman et al., 2021b; Roterman et al., 2021c). In the membrane environment, a distribution opposite to that representing the presence of a centrally located hydrophobic nucleus is expected. This is because the exposure of hydrophobicity is expected for a favorable entropy–enthalpy arrangement toward the environment and contact with the amphipathic membrane.

The presence of prefoldin in protein folding was deemed a means of providing an external force field for the folding protein. The FOD-M model quantifies the environmental change introduced by the presence of the introducing molecules with respect to the aqueous environment.

In experimental studies on prefoldin, it was found to be mainly involved in the folding of actins and tubulins. Therefore, representatives of these groups of proteins are also present in the analysis performed here. The aim is to demonstrate the status of their final, biologically active structural form.

The aim of this study is to test the option of interpreting the presence of prefoldin as a representative of the environment for stabilizing the structuring of a polypeptide chain whose folding process involves chaperonin (mainly of the Hsp40 group). Considering the characterization of prefoldin as a provider of an external force field for the stabilization of a structure different from that which would be obtained by the chain in an aqueous environment is intended to enable the simulation of the protein folding process *in silico*, taking into account a modified external field expressing the presence and effect of external factors. The activity of prefoldin is treated as guiding the step between the ribosome- and chaperonin-assisted steps of protein folding.

## 2 Results

The object of the analysis is the set of three proteins. Two of them represent the cytoskeleton construction: actin and tubulin. The third one is prefoldin—the protein that participates in the folding process of the two mentioned proteins.

### 2.1 Prefoldin-assisted products

Prefoldin activity has traditionally been linked with participation in actin and tubulin folding (Geissler et al., 1998; Vainberg et al., 1998). The representative structures of these proteins were assessed to determine their status in using the FOD model. This seeks to answer the question of whether the final form of the structure of these proteins reproduces a micelle-like arrangement or how the structure of these proteins is far from such an arrangement. Analysis of the status of the actin and tubulin protein representatives also answers the question of whether the structure they represent can be a product of the polar force field of water directing the folding process toward a micelle-like form. Experiments prove that the final forms of these proteins are obtained with the participation of the corresponding chaperonin (Siegert et al., 2000). Nevertheless, there is also a contribution from the preparatory step in which prefoldin mainly plays a role. This preliminary step in the folding process of actin and tubulin proteins, with the participation of prefoldin, is aimed at eliminating the influence of the aqueous environment on the structuring of these proteins.

This group of actin proteins is represented by *Caenorhabditis elegans* mg-atp actin in the complex with the human gelsolin segment (PDB ID 1D4X) (Vorobiev et al., 2003).

**FIGURE 1:**
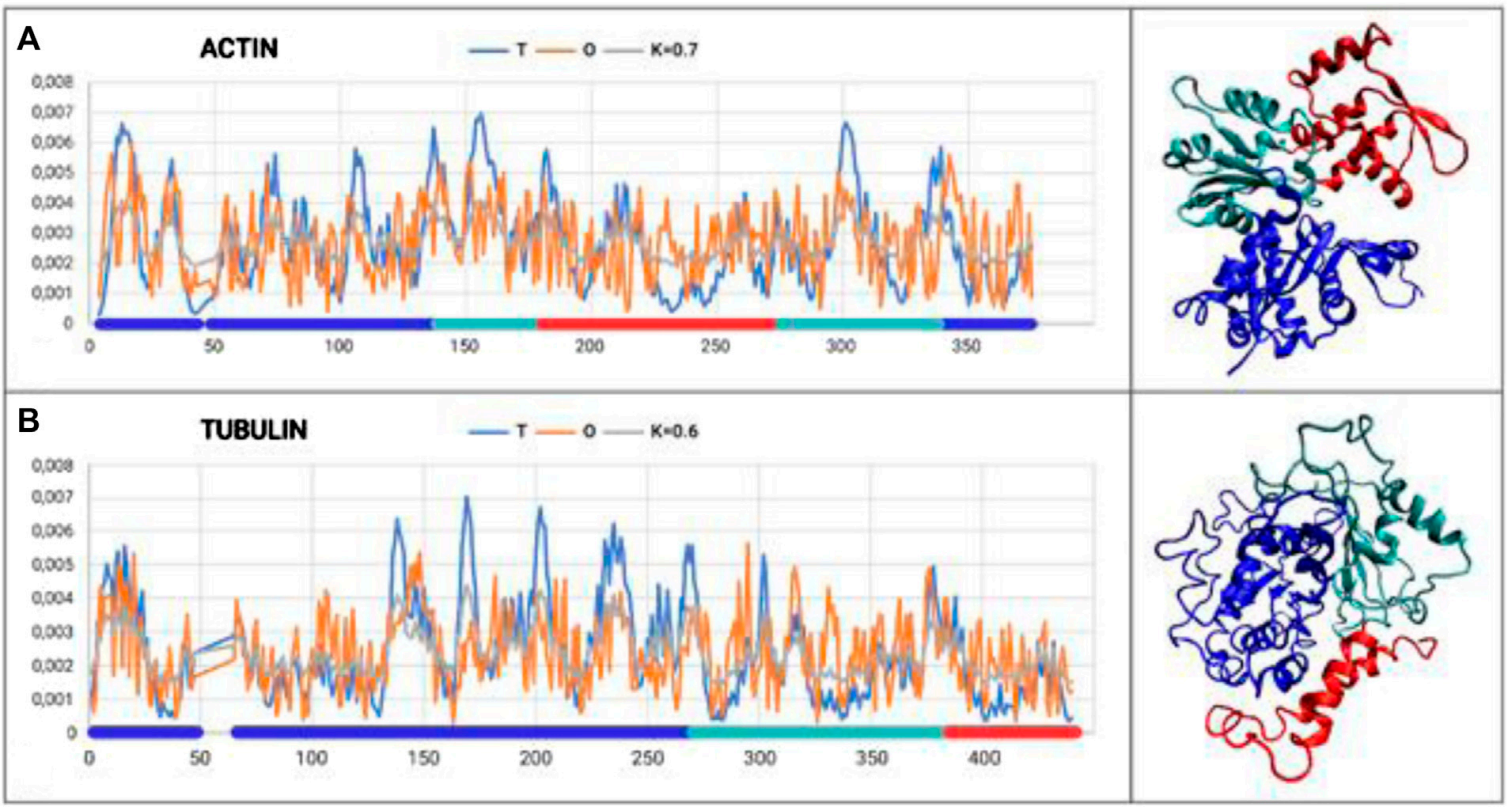
Set of T, O, and M profiles for monomers of actin **(A)** and tubulin **(B)** along with 3D presentation. Domains distinguished in colors according to Tables 1, 2.

The analysis is restricted to the A-actin chain, where three domains were distinguished according to the CATH classification (Figure 1A).

The results provided in Table 1 with the RD and K parameters describing the status of the actin molecule suggest a far from micelle-like distribution. Values of RD = 0.606 and K = 0.7 imply a folding process in the presence of an external force field different from the aqueous environment. When analyzing the status of the individual domains, it can be concluded that Dom1 and Dom2 viewed as individual structural units show a micelle-like arrangement. Dom3, on the other hand, clearly deviates from such a status. Particularly, Dom3, as a component of the whole molecule, shows a significantly different arrangement with respect to the micelle-like arrangement. Theoretically, Dom1 and Dom2 can fold in an aqueous environment showing the presence of a hydrophobic nucleus. In contrast, the mutual arrangement of the domains ultimately showing a significant deviation from the micelle-like arrangement implies the need for the presence of an external field different from the aqueous environment—the values for the structure of the complete chain are high—both RD and K.

**TABLE 1:** Summary of RD and K-parameter values for actin and the domains distinguished in its structure. Values are submitted for domains as components of the entire chain and considered individual structural units. The status of residues involved in the inter-chain interaction (P-P) and not involved in the interaction (No P-P) is given in brackets. Domains distinguished by colors are also shown in Figure 1A.

Actin	Fragment	RD (P-P/No P-P)	K
1D4X Chain A		0.606	0.7
Part of the complete chain			
Domain 1 (blue)	(4–137) (339–373)	0.556 (0.338/0.555)	0.5
Domain 2 (cyan)	(138–179) (272–338)	0.576 (0.458/0.592)	0.4
Domain 3 (red)	(180–271)	0.712 (−)	1.2
Individual domain			
Domain 1	(4–137) (339–373)	0.485 (0.261/0.487)	0.3
Domain 2	(138–179) (272–338)	0.450 (0.449/0.454)	0.3
Domain 3	(180–271)	0.608	0.6

Prefoldin participates in the folding of the other protein, which is tubulin. Tubulin is a protein that is a major component of the eukaryote cytoskeleton (Bera and Gupta, 2022). This group is represented in the present analysis by a single chain A (PDB ID—1FFX) (Gigant et al., 2000) that is part of a complex termed the stathmin-like domain. The complex is made up of alternating linearly aligned chains A, C, B, and D, with chains A and C (tubulin α1) and B and D (tubulin β) being identical in pairs. These four chains are linked together via the E chain, which (mainly in the form of a helix) holds the four chains in a stathmin-like form.

The chain structure of the tubulin under consideration shows a status considerably divergent from the micelle-like form (Table 2). High RD values > 0.5 coupled with relatively high K values suggest the presence of a system far from the micelle-like arrangement (Table 2). This implies that both the folding of the whole chain and its constituent domains do not take place with the involvement of the aqueous environment. This is confirmed by the summary lists of T and O profiles (Figure 1B), which reveal the structural differentiation of the individual domains and, at the same time, show a distribution different from the micelle-like form.

**TABLE 2:** Summary of RD and K-parameter values for tubulin chain A and domains deemed chain components (PDB ID—1FFX). The bottom part of the table quotes the RD and K-values for domains viewed as individual structural units (3D Gaussian function generated for each domain individually). The status of the residues interacting with the other chain (not examined here but available in the 1FFX file) is also given in brackets. Domains distinguished by colors are also shown in Figure 1B.

Tubulin	Fragment	RD (P-P/No P-P)	K
1FFX–Chain A		0.571 (0.678/0.570)	0.6
Domain as part of the chain			
Dom1 (blue)	(1–268)	0.509 (0.451/0.521)	0.4
Dom2 (cyan)	(269–383)	0.574 (−)	0.6
Dom3 (red)	(384–440)	0.653 (0.684/0.621)	0.9
Individual domain			
Dom1	(1–268)	0.501 (0.603/0.507)	0.4
Dom2	(269–383)	0.523 (−)	0.4
Dom3	(384–440)	0.653 (0.366/0.668)	0.7

Interpretation of both proteins folded with prefoldin suggests the presence of a structure that differs from the micelle-like system with respect to hydrophobicity distribution. Values of K > 0.5 suggest a significant contribution from a non-hydrophobic environment.

RD values < 0.5 show the status of individual domains viewed as independent structural units in actin, suggesting that these chain fragments may fold on their own as a result of the presence and orientation originating in the aqueous environment. The final—function-related—arrangement, however, shows the need for specific targeting of the target-oriented folding process for the biological activity of the final product. Viewing Dom2 in actin as folded according to the micelle-like model despite RD < 0.5 is questionable due to the composition of this domain. This is because it is composed of segments with different locations (the chain of this domain is not a continuum). The formation of such a domain probably requires the involvement of an external factor directing the structuring of a domain composed of distant chain segments (in the sense of an I-order structure).

Moreover, folding with a clear orientation to expose residues prepared to interact with other chains (the proteins represent the IV-order structure) can most likely take place precisely with the involvement of an external factor. The complex of prefoldin with the folding protein is not accessible. Therefore, it is difficult to identify a specific role for the involvement of prefoldin in the folding process.

The presence of a single domain with high RD and K values (Dom3 in actin and Dom3 in tubulin) constitutes a characteristic feature in both structures discussed here, which may be the result of targeting orientation. The status of the residues involved in interactions with other chains (IV-order structure) differs from the micelle-like model. In many complexes, the residues involved in the inter-chain interaction show an increased level of hydrophobicity (RD for no P-P is elevated), which supports complexation based on the formation of a hydrophobic nucleus common to the complexed chains (RD for residues not involved in inter-chain interactions is low). An example here is the analysis of the structure of dystrophin, where a jointly formed domain by segments of two independent chains forms a structure with a common hydrophobic nucleus with a structure highly similar to a micelle-like system (Banach et al., 2020). For the proteins discussed here, the status of the residues exposed for inter-chain interactions does not show incompatibility with the micelle-like arrangement, ruling out complex formation based on the structure of a common hydrophobic nucleus.

It should be noted that prefoldin does not impose a final structure on the folding protein. This is determined by a process involving chaperonins. Nonetheless, the role of prefoldin as a factor that eliminates the involvement of the aqueous environment at an early stage of protein folding does play a significant role in this process.

The conclusion drawn from this part of the analysis identifies actin and tubulin molecules as representing a micelle-like far-field structure. This structure was obtained with Hsp40, although the contribution of prefoldin prevented chain folding according to the model expressed by the 3D Gaussian function with respect to hydrophobicity distribution.

**FIGURE 2:**
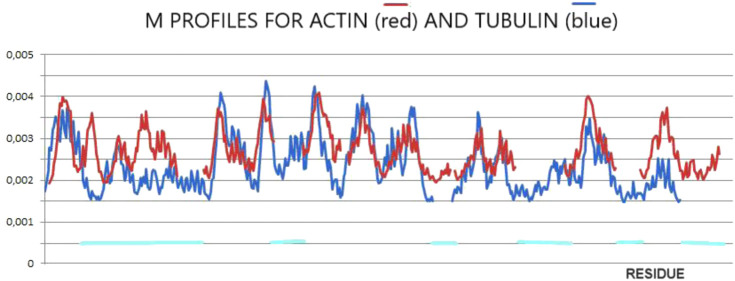
M-distributions for actin (red) for K = 0.7 and tubulin (blue) for K = 0.5 visualizing similarity interpreted as a possible common (for both proteins) influence of the external force field of pre-folding origin. The horizontal lines distinguish fragments of different statuses in actin in comparison with tubulin.

The final structures of actin and tubulin are fixed in chaperonin. However, one can speculate as to the prefoldin participation in the folding process of both these proteins comparing their M profiles (Figure 2). The optimal hydrophobicity profiles appear similar (to a limited degree) for those two proteins. The fragments with comparable M_i_ levels appear to represent the discordant status in native forms (Figures 1, 2). It suggests the aim-oriented fixation of the structural forms of these chain fragments.

### 2.2 Structure of prefoldin

The structure of prefoldin (GIMc—PFD) is available in the form of complexes with the ring-shaped chaperonin TRiC/CCT: the PDB ID—6NRB (chaperonin class 2), 6NRC (chaperonin class 3), 6NRD (chaperonin class 4), 6NR9 (chaperonin class 5), and 6NR8 (chaperonin class 6)*.* The structures represent different forms of prefoldin in complex with the chaperonin that is the target for the transport of the folded protein by prefoldin.

The structures are distinguished as follows: beta-sheets, yellow; sections representing no ordered secondary form, green; and helical sections showing “fringes”, maroon.

Yellow and green sections collectively referred to as “stem” in jellyfish construction. A set of helical sections are referred to as “tentacles.”

**FIGURE 3:**
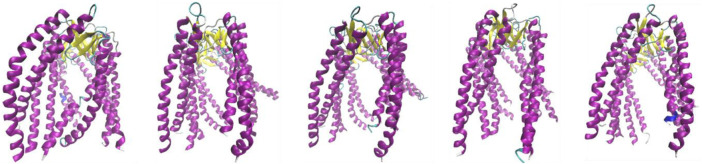
Structures discussed in this work. Subsequent images represent class 2 (PDB ID—6NRB), class 3 (PDB ID—6NRC), class 4 (PDB ID—6NRD), class 5 (PDB ID—6NR8), and class 6 (PDB ID—6NR9). The variation is due to the different forms of complexation toward TRiC/CCT.

Figure 3 shows the specific arrangement of six chains referred to as jellyfish construction, where the arrangement involving the β-sheet constitutes the “stem,” and the loosely flowing down helical sections are referred to as “tentacles.” Structural diversity was assessed in this study for the entire PFD complex (six chains) but also for its individual parts—chains considered individual structural units. Furthermore, the common part made up of the β-structure (stem) and a set of 12 helical segments (tentacles) was also considered individual structural units.

This means to assess the structure of the overall complex (six chains), a 3D Gaussian function is generated, and the degree of adaptation of the hydrophobicity distribution to a system with a centrally located hydrophobic nucleus is sought. The degree of presence of such a centric nucleus (compatibility of the O-distribution with the T-distribution) is expressed by the value of the RD parameter. The contribution of other, non-aqueous modification factors to the environment is also assessed by the value of the K-parameter. The T-distribution is modified by the K-parameter to a form—the M-distribution—that most closely reproduces the observed O-distribution.

Such an assessment was carried out for successive complexes (six chains together for each class) and for individual chains that were considered individual structural units.

Table 3 A–E summarize the values of the RD and K parameters. The RD parameters quoted in the Complex column describe the status of the entire complex (PFD—six chains) with respect to the idealized system—conforming to a 3D Gaussian distribution. The same column also gives the status of each chain as a component of the complex.

**TABLE 3:** A–E RD and K-parameter values for the complex and components. Values communicate the status of a chain within the complex (columns in the Complex title) and the status as an individual structural unit. The status of the part showing β-sheet structure (“stems”), and the part made of helical fragments (“tentacles”) and non-helical fragments (“no helices”) is also shown. Chain numbers highlighted * represent chains without contact (interaction) with chaperonin.

A				
*6NRB Class 2*	Complex		Individual chain	
6NRB	RD	K	RD	K
Complex	0.599	0.7		
Chain 1*	0.578	0.8	0.716	1.0
Chain 2*	0.548	0.5	0.699	1.2
Chain 3*	0.540	0.4	0.666	0.9
Chain 4	0.654	1.0	0.699	1.5
Chain 5*	0.614	0.6	0.753	1.1
Chain 6	0.629	1.0	0.653	0.8
Helices	0.647	1.2		
Beta-sheets	0.561	0.3		
No helices	0.517	0.3		

B				
*6NRC Class 3*	Complex		Individual chain	
6NRC	RD	K	RD	K
Complex	0.655	1.0		
Chain 1	0.629	1.1	0.731	1.5
Chain 2*	0.596	0.7	0.731	1.5
Chain 3*	0.643	0.6	0.704	1.1
Chain 4	0.660	1.2	0.710	1.7
Chain 5	0.558	0.5	0.719	1.0
Chain 6	0.632	1.1	0.650	0.8
Helices	0.700	1.5		
Beta-sheets	0.568	0.4		
No helices	0.544	0.4		

C				
*6NRD Class 4*	Complex		Individual chain	
6NRD	RD	K	RD	K
Complex	0.600	0.7		
Chain 1	0.628	0.8	0.726	1.3
Chain 2	0.565	0.8	0.732	1.4
Chain 3*	0.590	0.5	0.750	1.2
Chain 4	0.618	0.8	0.714	1.4
Chain 5	0.583	0.5	0.734	1.0
Chain 6	0.566	0.5	0.624	0.7
Helices	0.658	1.1		
Beta-sheets	0.494	0.2		
No helices	0.456	0.2		

D				
*6NR9 Class 5*	Complex		Individual chain	
6NR9	RD	K	RD	K
Complex	0.666	1.1		
Chain 1	0.605	1.0	0.715	1.3
Chain 2	0.629	0.7	0.734	1.4
Chain 3*	0.703	0.7	0.772	1.3
Chain 4	0.674	1.3	0.734	1.6
Chain 5	0.592	0.7	0.750	1.2
Chain 6	0.629	1.1	0.646	0.7
Helices	0.716	1.7		
Beta-sheets	0.507	0.3		
No helices	0.524	0.4		

E				
*6NR8 Class 6*	Complex		Individual chain	
6NR8	RD	K	RD	K
Complex	0.622	0.7		
Chain 1	0.648	0.9	0.754	1.4
Chain 2	0.549	0.5	0.714	1.1
Chain 3	0.595	0.5	0.739	1.2
Chain 4	0.709	1.0	0.754	1.5
Chain 5	0.580	0.5	0.765	1.1
Chain 6	0.562	0.5	0.652	0.8
Helices	0.679	1.1		
Beta-sheets	0.451	0.2		
No helices	0.467	0.2		

The status of an object with RD < 0.5 communicates a structuring consistent with the 3D Gaussian model.

An RD value > 0.5 shows a hydrophobicity distribution different from the centric nucleus and polar surface. An RD value close to 1.0 indicates almost complete negligence of the impact of the aqueous environment directing the chain structuring toward a micelle-like distribution with a centrally located hydrophobic nucleus and a polar surface.

The values of the K parameter, on the other hand, determine the degree of field modification expressed by the 3D Gaussian function. The higher the value of K is, the greater the contribution of non-water factors to structuration will be. A high value of K implies a significant modification of the 3D Gaussian function changing a system with a centric nucleus and a polar surface to a system where hydrophobicity is, correspondingly, not concentrated in the center of the system.

The column named Individual chain gives an analogous set of parameters for each chain viewed as an individual structural unit.

In Table 3 A–E, PFD chains that are not in contact with TRiC/CCT are also identified. Contacts between a chain and TRiC/CCT can notably affect its structuring.

The status of successive structural forms of the complexes is described by high RD and K values, showing a structuring different from that induced by the aqueous environment. RD values varying from 0.599 to 0.666 with K variation from 0.7 to 1.1 suggest the presence of a hydrophobicity distribution far from the idealized micelle-like arrangement (Table 3 A–E). The highest values of the parameters describing the status of the complexes occur for structures considered intermediates.

**FIGURE 4:**
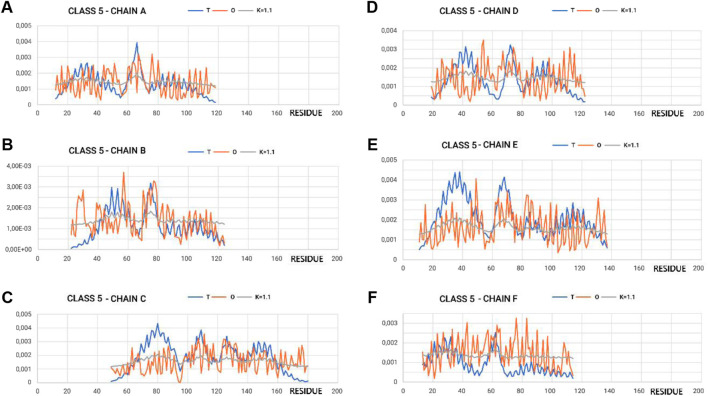
Profiles T, O, and M for appropriate K-values for chains in class 5 selected to represent the highest discordance with respect to hydrophobicity distribution, as expected in micelle-like organization. **(A–F)** according to chain identification.

Figure 4 shows the variation in hydrophobicity levels assessed for the complex. Systems with extreme status were selected for presentation.

**FIGURE 5:**
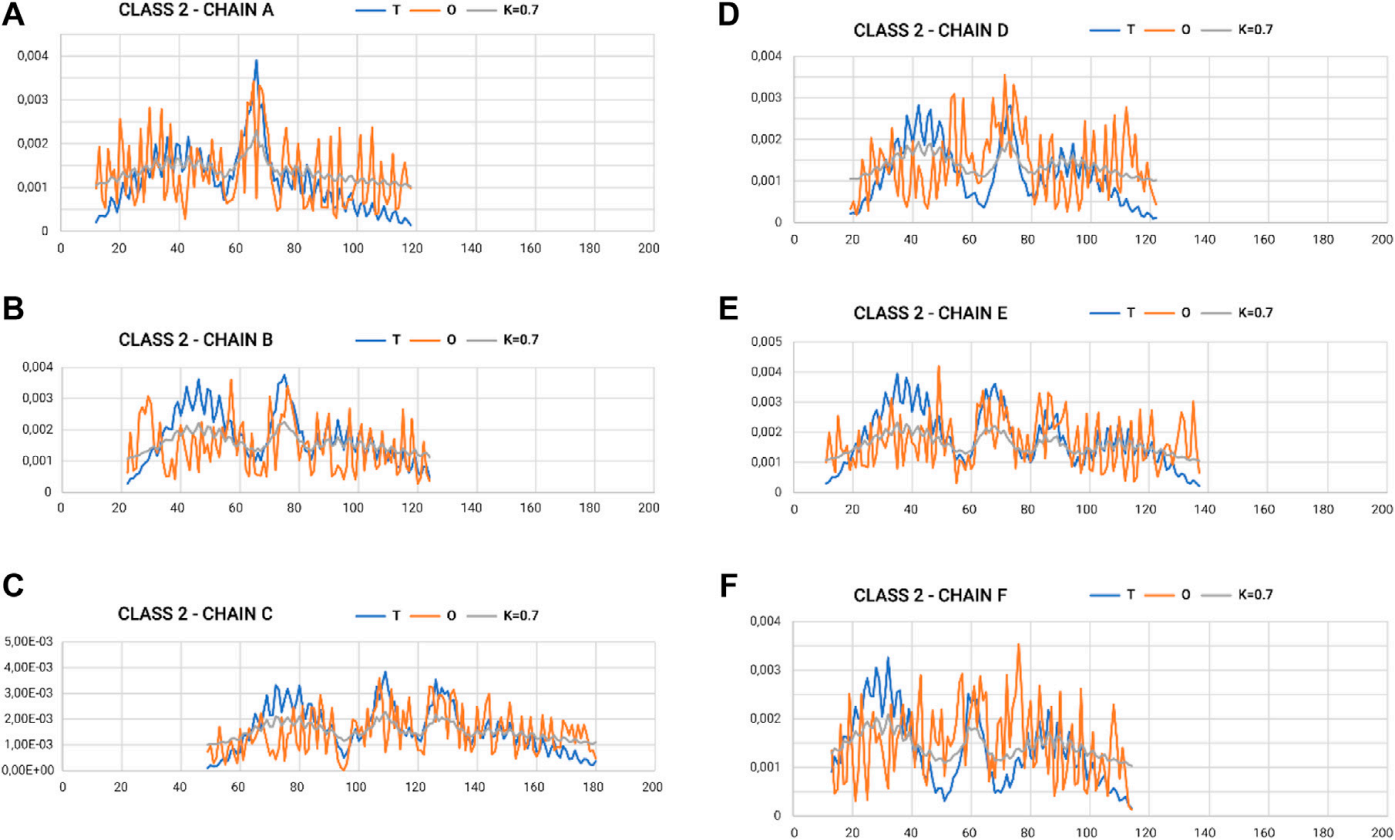
Profiles T, O, and M for appropriate K-values for chains in class 2 selected to represent the lowest discordance with respect to hydrophobicity distribution, as expected in micelle-like organization. **(A–F)** according to chain identification.

In the graphs of Figures 4, 5, where the profiles for the six chains are compared, one can see the central section showing the relatively high degree of adjustment of the O-distribution against the T-distribution for class 2 (sections 60–100) (red O-graphs—close to blue T-graphs). The middle section contains the β-structure distribution.

The proximity of the M-distribution to a straight line (distribution close to the R-distribution) suggests an almost complete disconnection from conditioning due to the presence of water. This is particularly evident in class 5 (Figure 4A). The M-plots are almost entirely close to the horizontal line. Interpretation of the results concerning the status of the PFD complexes suggests a structuring far from a micelle-like system. High values of K clearly communicate that the distribution of M is similar to that of R. This implies a structuring with no impact of the water environment, which directs the structuring toward a concentration of high hydrophobicity in the center. In this case, the distribution of hydrophobicity suggests almost no variation in the level of hydrophobicity within the complex.

### 2.3 Components of the complex

This section discusses the results concerning the status of the component parts of the complexes: the β-structural part (“stems”) and the arrangement of loosely spaced helical sections (“tentacles”). These separated parts were viewed as individual structural units, for which a 3D Gaussian function is defined.

**FIGURE 6:**
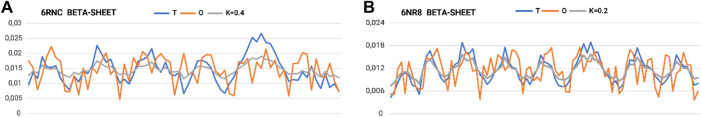
Set of profiles for β-sheets with **(A)** maximum and **(B)** minimum need for distribution modification attributable to the presence of an aqueous environment (outer field in the 3D Gaussian form).

The part referred to as “stems”—the middle part of the chains that together form the β-sheet system—is characterized by a set of profiles (Figure 6A). The two (Figures 6A, B) extreme sets of profiles T, O, and M for the highest value of K (0.4) and the lowest value of K (0.2) are summarized.

**FIGURE 7:**
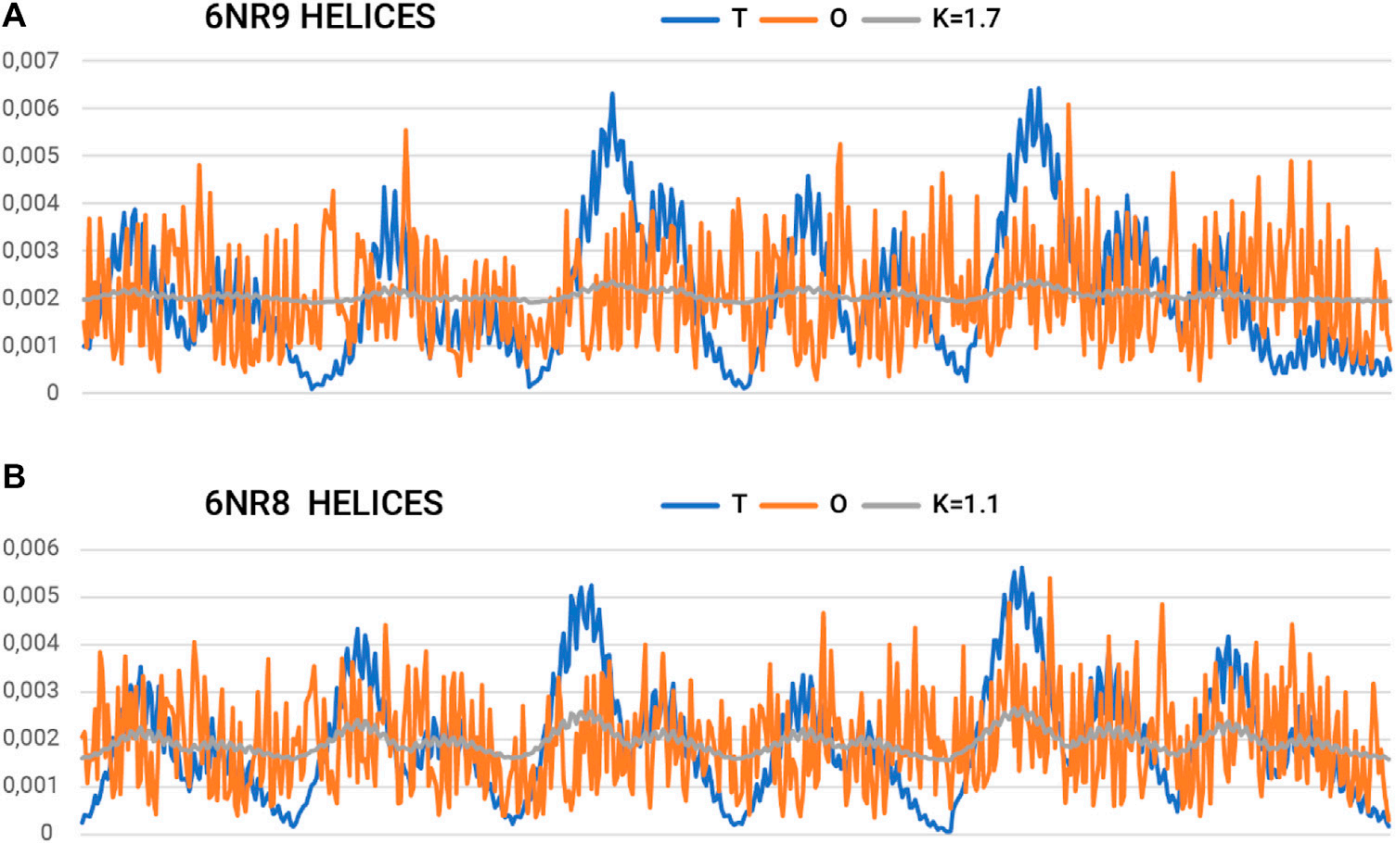
Summary of T, O, and K profiles for the “tentacles” part (helical sections) of the prefoldin expressing **(A)** the highest and **(B)** the lowest need for field modification.

The very low K-values for the “stems” part suggest their local structural approach toward an idealized micelle-like arrangement (Figure 7; Table 3 A–E). The “stems” arrangement represents a status suitable for an aqueous environment with a centrally located hydrophobic core, which makes this arrangement stable (the hydrophobic core, along with the disulfide bonds, is considered a stabilizing factor for the III-order structure). Thus, this part of the structure of the complex is probably responsible for stabilizing the system in the aqueous environment in which prefoldin acts.

On the other hand, a separate characteristic is represented by the system of 12-helical sections (“tentacles”). Here, the determined RD and K-values are very high. Values of K > 1.0 imply a material degree of maladaptation to the aquatic environment.

The summary list of profiles for the helix arrangement (Figure 7) reveals an ordering of hydrophobicity close to the distribution of R. This implies a significant departure from the micelle-like arrangement and thus represents a structure that does not follow the rules of the aqueous environment. This description applies to the structure of the helical parts. However, the status of this part may be considered a specificity of the field that a set of chains represents. A transported polypeptide chain complexed with the helical part of a prefoldin is subject to the ordering represented by this part. Hence, the arrangement of helices can be viewed as an external field with specificity remote from the aqueous environment. The presence of the “tentacles” part for the transported protein represents an environment—an external force field to which the transported chain presumably adapts. Indeed, the transported polypeptide chain is imposed with an ordering that is compatible with the arrangement present in the prefoldin. Comparable Oi values for almost all residues in the helical parts create an environment in which water does not express its specificity. Consequently, the transported chain is influenced by a system that induces a uniform distribution of hydrophobicity.

### 2.4 Status of individual chains

Furthermore, the structure of individual chains must be assessed. Such an analysis provides information on the contribution of each chain to the construction of the prefoldin, thus contributing to an external force field with respect to the transported protein.

The status of individual chains determined by high RD values points to structuring that cannot be obtained as a result of folding involving an external force field from the aqueous environment.

**FIGURE 8:**
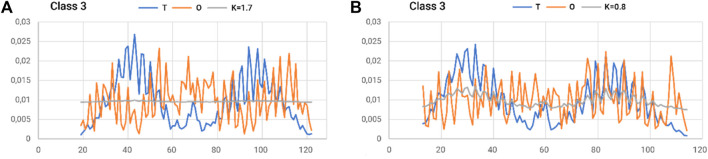
Summary of T, O, and M profiles for **(A)** class 3–chain 4–high K and **(B)** class 3–chain 6–lowest K-value for a set of chains deemed individual structural units. A value of K = 0.8 also indicates a high contribution of non-aquatic factors in shaping the structure of a chain.

The status of the individual chains analyzed in this subsection was determined while addressing each chain as an independent structural unit (Figure 8). In other words, a 3D Gaussian function was generated for each chain. Such an analysis is intended to present the characteristics of the chain folding process. High values of the RD parameters often above 0.7 including K > 1.0 suggest a structuring distant from that generated by the aqueous environment. This is visualized by a set of profiles for chains of extreme status (Figure 5).

To summarize the section on the analysis of the prefoldin structure, it should be noted that it is a structure with characteristics far distinct from the micelle-like model. The degree of distinctness from the structure with a central hydrophobic nucleus is significant. In other words, the complex was formed according to a scheme that does not conform to the aqueous environment.

Nevertheless, the part containing the β-structure appears to represent a status with a centrally located hydrophobic nucleus. In contrast, the part of the “tentacles” made up of helical sections features very high differentiation toward the micelle-like model.

At the same time, the characteristics of prefoldin are regarded as providing an external force field for the transported protein. Particularly, the part accessible to the transported chain provides an external field with an almost uniform distribution of hydrophobicity. This means that the transported chain resides in an environment that is isolated from the impact of the aqueous environment, thus preventing structuring that is not expected for the protein. The structuring that forms a central hydrophobic nucleus with a polar surface is eliminated.

## 3 Discussion

The aqueous environment is natural for most proteins. It is, therefore, a natural process for bipolar molecules, such as all amino acid residues with varying ratios of polarity to hydrophobicity, to seek to structure the chain in a micelle-like form. This implies a natural tendency to isolate the hydrophobic residues in the center and expose the polar residues on the surface. Groups of proteins meeting the criteria for micelle-like structuring have been identified (Banach et al., 2020). Furthermore, it has been shown that the vast majority of domains viewed as individual structural units also represent this type of ordering (Banach et al., 2020). These groups of proteins are characterized by K = 0 or very close to it (K < 0.4). The presence of a cell membrane environment with different characteristics impacts the structuring of proteins in this active environment, directing the structuring process differently by exposing hydrophobic residues on the surface and generating low polar packing in the center (particularly ion channels). The FOD-M model allows quantitative evaluation of the degree of remoteness for aqueous environments.

The polar environment (water–K < 0.5) and amphipathic environment (membrane–0.9 < K < 1.5) expressed by the high value of the K-parameter and characterized with it reflect the specificity of these two external force fields, directing the folding process toward a system compatible with the specificity of the external field. These two examples of extremely opposing environments (water and cell membrane) were taken as the basis for extending this interpretation to other environments. This was obtained for the periplasm environment, where K = 0.6 (Roterman et al., 2022).

The K-value can be interpreted as the structural specificity of the product expressing the influence of the environment. However, the structure of a certain protein can generate the external force field for the polypeptide chain transported by prefoldin as it is in the case discussed in this paper. The environment created by the helical fragment system of prefoldin was covered in the same way, considering it an external force field for the transported protein. The presence of the prefoldin environment does not affect the final form of the folded protein but prevents micelle-like structuring, which could occur to a greater or lesser extent if the chain was subjected to just such an external force field.

Reports of the appearance of amyloid forms when prefoldin is deactivated suggest the importance of the environment for protein folding. The amyloid form of α-synuclein appears in an aqueous environment (low K for the amyloid form of this fibril), while the natural environment (reflected in the cryo-EM experiment as a micelle complex) dictates the structuring of the native form with a high K-value. This high K-value is an assessment of the influence of the environment for the stabilization of the functional form of α-synuclein interacting with the axon terminals of presynaptic neurons (Roterman et al., 2021d). The presence of a target is the natural environment for α-synuclein, which retains its structuration with a high K-value by interacting with this target. When moving into an aqueous environment, α-synuclein adjusts its structuration to a micelle-like form with a significantly lower K-value (RD < 0.5).

The relationship of the native form of transthyretin to its amyloid form is assessed in contrast. The native forms of transthyretin with a low K-value (aqueous environment) in the amyloid form show structuring with a high K-value. This implies that the formation of the amyloid form is induced by a change in the characteristics of the environment (introduction of a factor that disrupts the natural environment for the activity of this protein including shaking, in particular).

**FIGURE 9:**
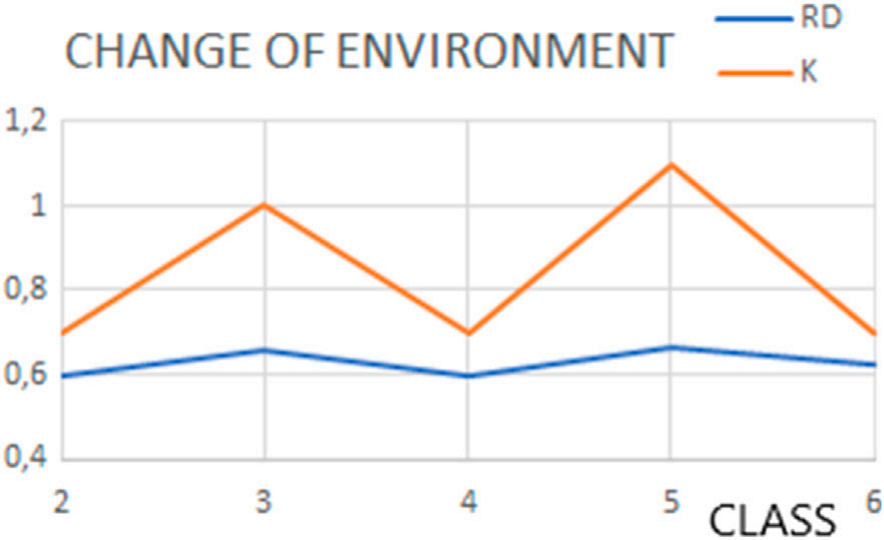
Changes in status (expressed by the RD and K values) of structures representing classes as classified by Gestaut et al. (2019), representing pulsating steps during the transport.

The changes in the prefoldin status (Figure 9) may be treated as periodic pulsation, introducing step-wise different external conditions for the folded protein.

### 3.1 Future development

Future application of observations presented in this paper is focused on two issues: protein folding simulation *in silico* and assessment of other components accompanying the folding process in the classification in the K-scale.

The introduction of an external force field in the folding simulation seems to be critical. The standard force fields applied in the protein structure prediction represent the averaged form of the external force field (parameters applied for force field expression), or the representation in the form of a box with an appropriate number of water molecules appears not to be sufficient. Introduction of a modified external force field (expressed by the M-profile) is assumed to direct the folding process toward the not necessarily micelle-like construction of hydrophobicity distribution. The assessment of an external force field in the K-scale applied to chaperone (Roterman et al., 2023) and chaperonin (Roterman et al., 2023) opens the possibility to simulate the folding process in a specific environment influencing structuralization (Roterman and Konieczny, 2023). In addition to the general influence of external conditions (mainly based on polarity/hydrophobicity changes), the contribution of reactive oxygen species (ROS) and the presence of certain divalent cations (mainly Zn^2+^, Fe^2+^, and Cu^2+^) in the media to promote the amyloid fibril formation should not be neglected (Cheignon et al., 2018).

## 4 Materials and methods

### 4.1 Data

The object of the analysis is prefoldin, the structure of which is available in PDB in several (five) forms that this protein adopts in complex with chaperonin (PDB IDs: 6NR8, 6NR9, 6NRB, 6NRC, and 6NRD) (Gestaut et al., 2019). The prefoldin structures available in the aforementioned files represent diverse forms of interaction with ring-shaped chaperonin TRiC/CCT (Gestaut et al., 2019).

Two proteins, actin (PDB ID—1D4X) (Vorobiev et al., 2003) and tubulin (PDB ID—1FFX) (Gigant et al., 2000), were also analyzed. The aim of this analysis is to assess the native, biologically active structure of these proteins viewed as a product of folding involving external factors (prefoldin and chaperonin) (Roterman et al., 2023; Roterman and Konieczny, 2023).

### 4.2 The model used—FOD-M

This model, described in the literature in numerous papers (Konieczny et al., 2006), is presented here in a short version to facilitate the interpretation of the results obtained.

The model, referred to as FOD, assumes that the hydrophobicity distribution in the protein has a form that can be expressed by a 3D Gaussian function Eq. (1).

$$\tilde{H}t_{j}=\frac{1}{\tilde{H}t_{sum}}\exp\left(\frac{-{\left(x_{j}-\bar{x}\right)}^{2}}{2\sigma_{x}^{2}}\right)\exp\left(\frac{-{\left(y_{j}-\bar{y}\right)}^{2}}{2\sigma_{y}^{2}}\right)\exp\left(\frac{-{\left(z_{j}-\bar{z}\right)}^{2}}{2\sigma_{z}^{2}}\right).$$ (1)

This function spans the body of the protein (the shape of the function is adapted to the size of the protein by means of appropriately chosen values of σ_X_, σ_Y_, and σ_Z_). The value of this function assigned to the position of the effective atom (the averaged position of the atoms comprising relevant amino acid residues) expresses the level of hydrophobicity idealized—Ti assuming that the distribution corresponds to a micelle-like system with a centrally located hydrophobic nucleus and a polar surface. In contrast, the actual status of an amino acid residue in a protein is the result of inter-amino acid residue hydrophobic interactions—Oi. The value of Oi depends on the distance between the interacting amino acid residues and their intrinsic hydrophobicity Eq. (2). A function proposed by Levitt (1976) was used to determine the Oi value.

$$\tilde{H}o_{j}=\frac{1}{\tilde{H}o_{sum}}\sum_{i=1}^{N}\left(H_{i}^{r}+H_{j}^{r}\right)\left\{\begin{array}{l} \left[1-\frac{1}{2}\left(7{\left(\frac{r_{ij}}{c}\right)}^{2}-9{\left(\frac{r_{ij}}{c}\right)}^{4}+5{\left(\frac{r_{ij}}{c}\right)}^{6}-{\left(\frac{r_{ij}}{c}\right)}^{8}\right)\right]\text{ for }r_{ij}\leq c\\ 0\text{ for }r_{ij}>c \end{array}\right..$$ (2)

The degree to which the O system (distribution under study–P in Eq. 3) reflects the T-system (reference distribution Q in Eq. 3) can be assessed by comparing the Oi and Ti distributions. To this end, the divergence entropy D_KL_ introduced by Kullback and Leibler (1951) was used.

$$D_{KL}\left(P|Q\right)=\sum_{i=1}^{N}P_{i}\log_{2}\frac{P_{i}}{Q_{i}}.$$ (3)

The D_KL_ value calculated for the relationship of the O- versus T-distributions cannot be interpreted without the introduction of another reference distribution. A uniform distribution R was used, where each amino acid residue is assigned the same hydrophobicity level equal to Ri = 1/N (N being the number of amino acids in the chain). This distribution represents a system where a hydrophobic nucleus is absent. Before determining the D_KL_ value, each distribution must be normalized. By comparing the D_KL_ values for O against T (O|T) and O against R (O|R), one can identify the distribution against which the O-distribution is similar (D_KL_ (O|T)<D_KL_ (O|R) communicates the presence of a hydrophobic nucleus). In order to eliminate the use of two values to describe the same object, the parameter RD (Eq. 4) was introduced, which can be expressed as follows:

$$RD=\frac{D_{KL}\left(O|T\right)}{D_{KL}\left(O|T\right)+D_{KL}\left(O|R\right)}.$$ (4)

RD < 0.5 indicates the presence of a hydrophobic core.

**FIGURE 10:**
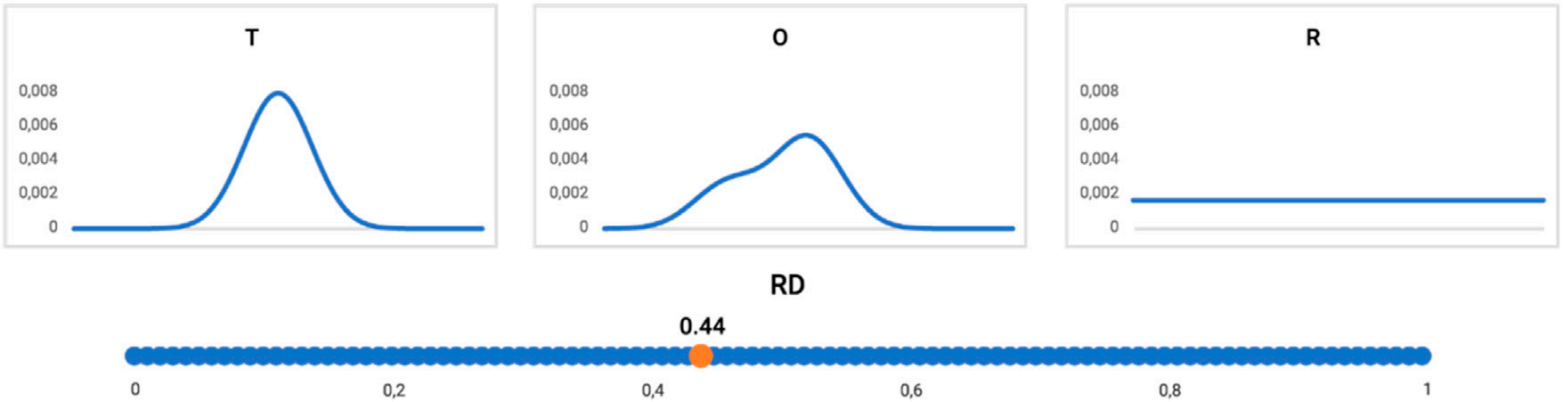
Profiles: T (upper left), O (upper central), and R (upper right) distribution (reduced to one-dimension). The RD value for this example = 0.44, as shown on the bottom line. The distribution O assessed according to the FOD model as representing the hydrophobic core (RD < 0.5).

The graphic presentation of the RD interpretation is shown in Figure 10.

Adopting a micelle-like distribution (hydrophobic center and polar surface) reproduces the system generated by the polar water environment as an external force field. Apart from the aqueous environment, proteins also act in a different environment—the membrane environment. Here, an exposure of hydrophobic residues on the surface—and for proteins acting as a channel of low hydrophobicity in the center of the molecule—is expected. This *status quo* can be described by a function complementary to the 3D Gaussian function (1–3DG) (3DG stands for 3D Gaussian function) (Eq. 5). In practice, the distribution is determined by the following formula:

$$T_{\mathrm{MAX}}-T_{i},$$ (5)

where *T*_*MAX*_ is the maximum value for the T-distribution determined for a protein, and *T*_*i*_ is the theoretical level for the *i-th* amino acid residue. It turns out that the distribution present in membrane proteins takes the following form:

$$Mi={\left[Ti+K^{*}{\left(T_{\mathrm{MAX}}-Ti\right)}_{n}\right]}_{n},$$ (6)

where the subscript “n” denotes normalization. This formula means that the structuring of the membrane protein is affected by the aqueous environment (function of *T*) modified by the contribution of the environment (*T*_*MAX*_–*T*_*i*_) to a degree determined by the value of K. This value is determined as min *D*_*KL*_ for the relation (*M|T*) (Eq. 3).

**FIGURE 11:**
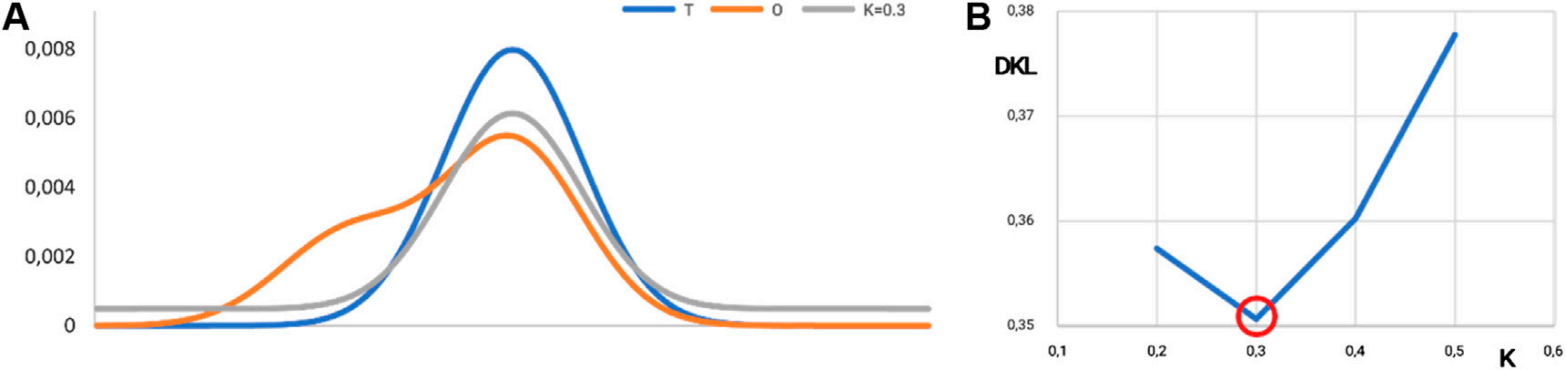
Visualization of the K-value. **(A)** Distributions: T, blue; O, orange; and M, gray for K = 0.3 in the discussed example. The best fit is shown in **(B)**. **(B)** Search for optimal K for the example given in Figure 10. The lowest value of D_KL_ for the (O|M) relation is reached for K = 0.3. The ray line on A represents the best fit of theoretical distribution with respect to O-distribution from Figure 10.

The interpretation of the example shown in Figures 10, 11 is provided as follows: the distribution of hydrophobicity (O), as it appears in the real example, differs from the idealized micelle-like distribution, as measured by RD = 0.44. It means the distribution is of a micelle-like category with a hydrophobic core and polar surface.

The value of the K-parameter (K = 0.3) means that the environment characteristics differ from the water environment in a low degree (K = 0.3 means that the environment is very close to the water environment).

The interpretation of the RD value is used to assess the status of the hydrophobicity distribution in a protein (against a micelle-like distribution—so the presence/absence of a hydrophobic core can be determined). The interpretation of the K-parameter determines the degree of field modification against the field created by polar water. A value of K = 0 is obtained for proteins described by Banach et al. (2020). Other soluble proteins in aqueous media represent values of 0 < K < 0.5, including numerous single-chain enzymes. A detailed analysis of proteins operating in periplasmic environments shows K = 0.7 (Roterman and Konieczny, 2023). In contrast, membrane proteins represent a spectrum of 0.7 < K < 1.5. Proteins with K > 3.0 were also identified (Roterman and Konieczny, 2023). The K-value is interpreted as a measure of the contribution of a deforming factor, modifying the aqueous environment leading to a structural form expressing a fit to the specific environment.

In this work, the need for complexation of the folding protein with prefoldin was considered a form of security to obtain a structure representing the effect of the aqueous environment, which is a structure with a centrally located hydrophobic nucleus and a polar surface. Thus, prefoldin was handled as a form of external force field for the folding protein.

An analysis based on the FOD-M model was also used to assess the structures of proteins known to be involved in prefoldin during their folding.

The FOD and FOD-M models are graphically presented here in detail. Hypothetical models are discussed to assist in the interpretation of the RD and K parameters, as introduced in the FOD-M model.

**FIGURE 12:**
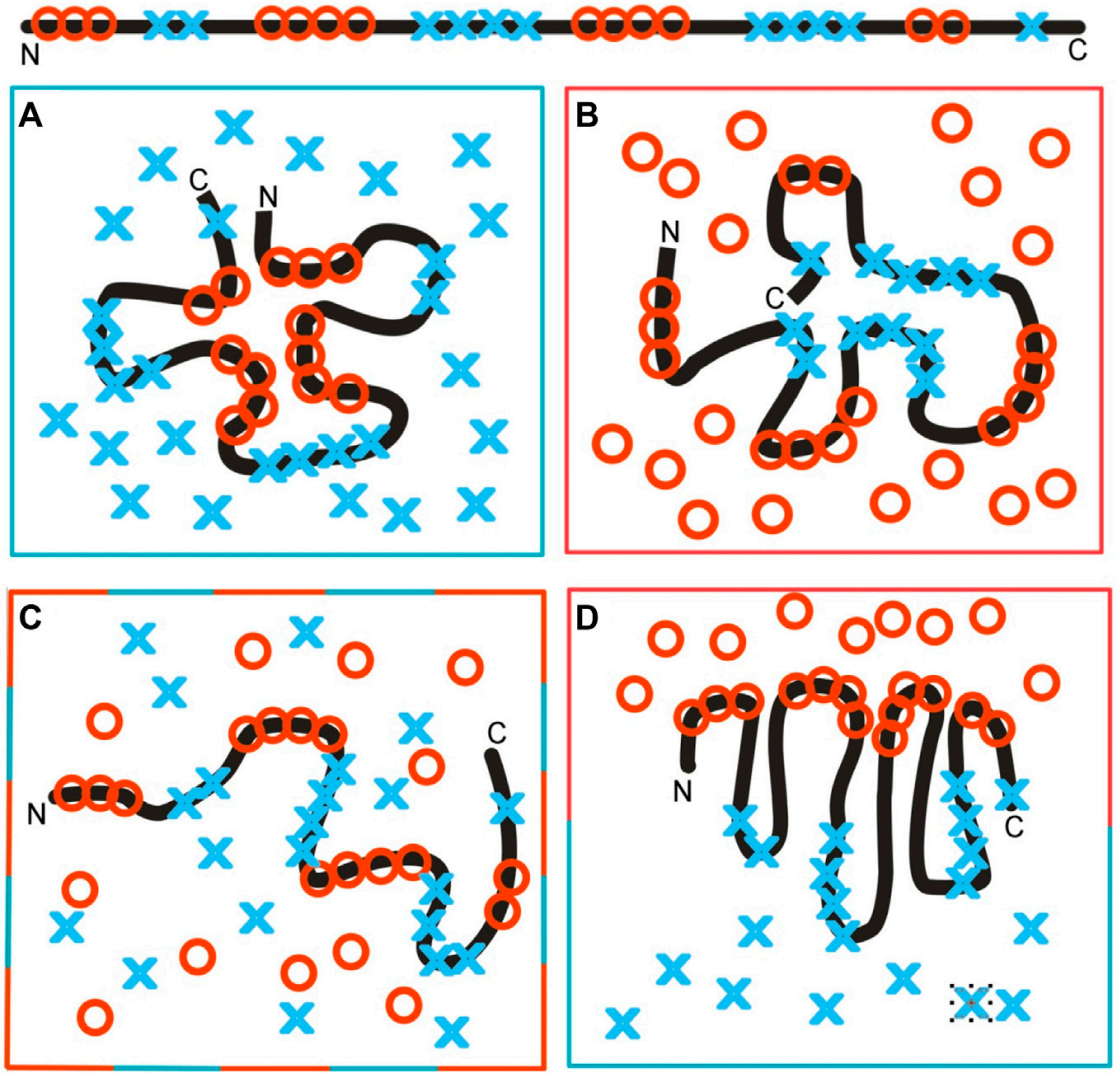
Structuralization of the polypeptide chain with hydrophobic and hydrophilic residues distributed as shown at the top. **(A)** Folding in a water environment, which directs the hydrophobic residues toward the central part, with hydrophilic residues exposed on the surface—hydrophobicity distribution expressed by the 3D Gaussian function (low RD and low K-values). **(B)** Folding in a hydrophobic (non-polar) environment, concentrating the hydrophilic residues in the central part of the hypothetical protein. Such a structure is described by high RD and high K-values, due to the absence of a hydrophobic core in the central part of the molecule. **(C)** Environment with a random distribution of polar (blue X) and non-polar (red O) molecules, leading to the unfolded form of the polypeptide chain. **(D)** Polarized environment with hydrophobic molecules in the associated form and distributed water molecules, leading to an ordered structural form, but one which is far from the 3D Gaussian hydrophobicity distribution, also having high RD and K-values.

The influence of the environment on the polypeptide chain folding (sequence with hydrophobic and hydrophilic residues, as shown on top of Figure 12), as dependent on the environment, is shown in Figure 12. Figure 12A illustrates directional folding in a water environment. The polar molecules of water (blue X) influence the folding of the polypeptide chain, with the concentration of hydrophobic residues (red O) in the central part a consequence of the exposure of polar residues on the surface. A protein molecule folded according to this model, thus, generates a hydrophobic core. The hydrophobicity distribution conforms to the FOD model; it can be expressed by a 3D Gaussian function. The value of the K-parameter is very low (close to zero, or K < 0.3). Proteins with a structure corresponding to these conditions are described in Banach et al. (2020). The domains, treated as individual structural units, represent states with low K-values (Sałapa et al., 2012).

The colors in frames represent the form of the local environment.

The opposite situation is represented in Figure 12B, which shows a hydrophobic environment (red O) causing the exposure of hydrophobic residues on the surface. The (hypothetical) protein may represent a kind of a hydrophilic core localized in the central part, with hydrophobic residues exposed on the surface. Theoretically, membrane proteins—especially the domains anchored in the membrane—represent such a state. The K-value is very high in such a case with significant participation of the 1–3D Gaussian function.

The same amino acid sequence in a mixed environment (Figure 12C) adopts a random, or rather unfolded, structural form. The polar (blue X) and non-polar (red O) molecules in the environment change positions dynamically and cause dynamic structural changes in the polypeptide chain. The structure shown in this diagram cannot be assumed to be stable, in contrast to those shown in Figures 12A, B.

In the presence of polar molecules, the hydrophobic molecules tend to minimize the contact surface, generating the associations, as shown in Figure 12D. The structure of the polypeptide chain can adopt a dipolar form, with the hydrophobic terminal directed toward the hydrophobic associate and the polar terminal directed toward the water environment. This structure can also be described by a high K-value, due to the absence of a centric concentration of hydrophobic residues—as is expected in the case of the 3D Gaussian function.

**FIGURE 13:**
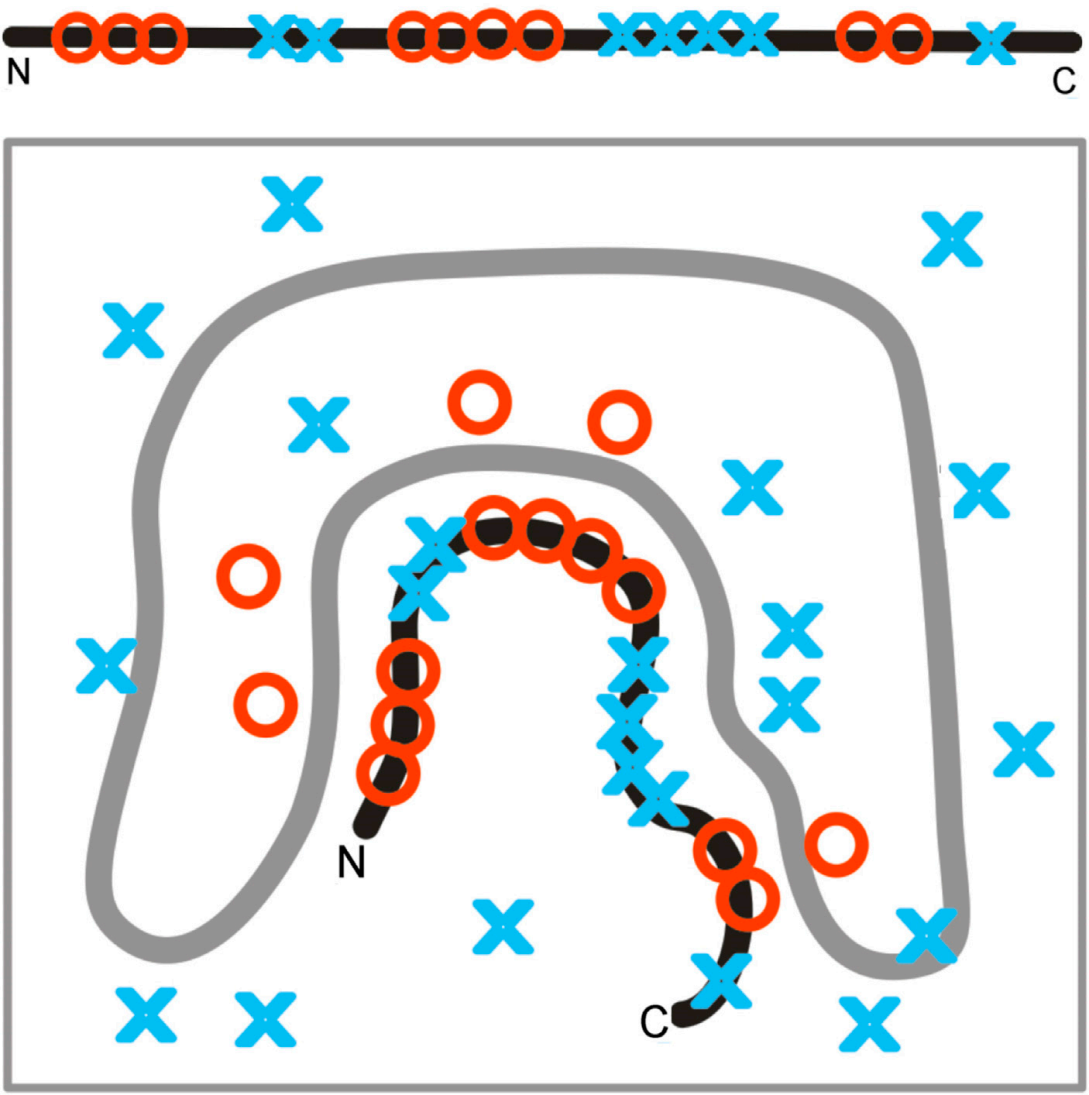
Fixed localization of polar and non-polar residues in prefoldin influences the structuralization of a polypeptide chain, far from attaining the 3D Gaussian hydrophobicity distribution. The gray line represents the structure of prefoldin, playing the role of a rigid frame for the transported polypeptide chain.

A random distribution of polar and non-polar molecules can be fixed by supplying a stable frame in which the polypeptide chain can fit, as shown in Figure 13. Fixed localization of polar and non-polar residues can be obtained in a protein-like prefoldin, which imposes a well-defined organization of the hydrophobic–hydrophilic distribution.

The structure of a polypeptide chain, as shown in Figure 13, represents a state with a relatively high K-value. Moreover, the K-value of the polypeptide chain structure may represent a state comparable to that of prefoldin, where the localization of polar/hydrophobic residues does not conform to the 3D Gaussian distribution.

The mathematical procedure enabling the calculation of the K-value can be used to assess the status of the hydrophobicity distribution in the folded protein (RD parameter) and the level of participation of an environment other than pure water (K-parameter). The environment is treated as an external force field for folding proteins, directing that process toward a structure of a complementary form with respect to hydrophobicity and hydrophilicity in the close neighborhood of the folding polypeptide chain (Figures 12, 13).

Structures with a well-defined hydrophobic core are stable (the SS-bonds and hydrophobic core are treated as stabilizers for III-order structuralization). Such structures are constructed when the stability of the system is necessary (Dygut et al., 2016). However, some proteins with some elasticity are expected to fulfill some biological functions. Elastin and tubulin are examples. These form complexes that are expected to exhibit some elasticity. The rigid structuralization of the complex appears disadvantageous in this case. For this reason, the structures of these proteins represent a hydrophobicity distribution relatively distant from that with a centric hydrophobic core.

An additional problem is related to the structuralization of prefoldin. The question is how does prefoldin become folded in such an unusual structural form? This issue is discussed in the final part of the main body of the paper. Single-chain folding is discussed in the main body of the paper, as is the construction of a six-chain complex.

### 4.3 Programs used

The potential user has two possible ways to access the program:

The program allowing the calculation of RD and T and O distribution is accessible upon request on the CodeOcean platform: https://codeocean.com/capsule/3084411/tree. The corresponding author can be reached to get private access to the program.

The application is implemented in collaboration with the Sano Centre for Computational Medicine (https://sano.science) and running on resources contributed by ACC Cyfronet AGH (https://www.cyfronet.pl) in the framework of the PL-Grid Infrastructure (https://plgrid.pl)—provides a web wrapper for the abovementioned computational component and is freely available at https://hphob.sano.science. The calculation time (even for long chains or complex proteins) is negligibly short using any model of laptop.

The VMD program was used to present the 3D structures (VMD, 2006; https://www.ks.uiuc.edu/Research/vmd/, accessed December 2022; Humphrey et al., 1996).

## 5 Conclusion

The structure of prefoldin—with which proteins requiring chaperone participation in the folding process—was considered a source of an external force field directing chain formation before the final folding process takes place. Prefoldin in the complex with the folding polypeptide chain is viewed as a factor eliminating the involvement of the aqueous environment, which would presumably direct the process toward a structure with a higher proportion of micelle-like ordering. The distribution of M (Figure 8) assumes the form of a straight line, which means almost complete independence from the polar external field originating from water (no hydrophobic nucleus is generated).

Thus, the analysis based on the FOD-M model suggests prefoldin is a provider of an external force field, preventing the chain from folding into a micelle-like structure, which would be formed in the aqueous environment. The application of the FOD-M model makes possible a quantitative evaluation to determine the degree of force field modification due to the presence of prefoldin (M-distribution) against a polar water environment (T-distribution). The degree of modification of the water-derived field is expressed with RD values ranging from 0.647 to 0.716 and, for the parameter K, a range of 1.1–1.7, indicating a considerable degree of modification.

Protein structures obtained with prefoldin show high RD and K values, which imply a folding of their chains different from the micellization process that occurs in aqueous environments. It is hypothesized that the introduction of a suitable external force field to simulate the *in silico* folding process may improve the prediction results as the environment acts as an active partner for non-binding interactions taking place within the molecule. Introducing the folding chain into an external force field expressed with an appropriate value of the parameter K (Eq. 6) is expected to direct its structuring toward a hydrophobicity distribution corresponding to the specificity of the environment.

The final status of proteins folded with the help of prefoldin appears comparable with the status of prefoldin itself. The assumption of the external force field delivering by prefoldin seems to be accurate. The dynamic changes in the prefoldin status (Figure 11) suggest the active participation in structure stabilization of the client protein.

## Data Availability

The raw data supporting the conclusion of this article will be made available by the authors, without undue reservation.
